# Association between circulating tryptophan metabolites and postoperative pain intensity in lumbar disc herniation surgery: An observational study

**DOI:** 10.1097/MD.0000000000043704

**Published:** 2025-08-01

**Authors:** Yunus Celik, Hale Aksu, Ferim Sakize Gunenc, Orhan Kalemci, Ceren Kizmazoglu, Pembe Keskinoglu

**Affiliations:** a Department of Anesthesiology and Reanimation, School of Medicine, Dokuz Eylul University, Izmir, Turkey; b Department of Neurosurgery, School of Medicine, Dokuz Eylul University, Izmir, Turkey; c Department of Biostatistics and Medical Informatics, Basic Medical Sciences, School of Medicine, Dokuz Eylul University, Izmir, Turkey.

**Keywords:** acute pain, kynurenic acid, microbiota, picolinic acid, quinolinic acid, tryptophan, xanthurenic acid

## Abstract

Gut microbiota is increasingly recognized for its involvement in pain modulation mechanisms, including visceral, inflammatory, neuropathic pain, and opioid tolerance. Although alterations in microbiota composition may affect host neuroimmune interactions, direct evidence in surgical pain contexts remains limited. This observational study explored associations between circulating tryptophan-derived metabolites and postoperative acute pain intensity in patients undergoing lumbar disc herniation surgery. Patients aged ≥ 18 years scheduled for lumbar disc herniation surgery were enrolled. Blood samples were obtained preoperatively and at 8 and 24 hours postoperatively to quantify tryptophan metabolites (picolinic acid, 3-OH kynurenine, anthranilic acid, kynurenine, quinolinic acid, kynurenic acid, and xanthurenic acid) via LC–MS/MS. Concurrent visual analog scale scores were recorded. Spearman correlation analysis was performed to evaluate associations between metabolite levels and pain scores. Thirty-seven patients (51.4% male, mean age 50.2 ± 11.8 years, body mass index 27.6 ± 2.9 kg/m^2^) were included. Significant perioperative changes were observed in picolinic acid (*P* = .001), kynurenic acid (*P* = .006), and xanthurenic acid (*P* < .001). Quinolinic acid levels correlated with 24-hour postoperative visual analog scale scores (*r* = 0.422; *P* = .009). Additional associations were identified between age and changes in picolinic acid (*r* = 0.357; *P* = .030), and between operation time and changes in 3-OH kynurenine (*r* = −0.359; *P* = .029). This exploratory analysis suggests that specific tryptophan metabolites, particularly quinolinic acid, may be linked to postoperative pain intensity and could serve as potential biomarkers in pain assessment. Future studies with direct microbiota profiling are needed to clarify the mechanistic pathways involved.

## 1. Introduction

Human microbiota, a complex and dynamic ecosystem of microorganisms residing throughout the body, plays a crucial role in maintaining health and contributing to disease. While the gut microbiota is best known for its role in digestion, emerging evidence highlights its broader impact on systemic physiological processes, including immune modulation, brain function, and pain regulation.^[1,2]^

Recent studies have drawn attention to the gut–brain axis and its role in modulating both acute and chronic pain through neuroimmune and neurochemical pathways.^[3,4]^ Microbial metabolites, particularly those derived from tryptophan metabolism, have been shown to influence pain signaling by interacting with receptors such as the aryl hydrocarbon receptor, thereby impacting central sensitization and inflammatory responses.^[5,6]^

Tryptophan, an essential amino acid, is metabolized by gut microbiota into a variety of bioactive compounds, including kynurenine, kynurenic acid, and quinolinic acid. These metabolites can either exacerbate or alleviate nociceptive transmission depending on their neuroactive properties.^[7,8]^ While several animal studies and mechanistic investigations suggest that microbiota-mediated tryptophan metabolism may affect pain thresholds, evidence in acute surgical pain contexts remains limited.^[9,10]^

One of the most widely used tools to assess pain intensity is the visual analog scale (VAS), despite its inherent subjectivity.^[11]^ Identifying objective biomarkers that correlate with subjective pain scores could enhance perioperative pain management strategies.

Currently, there is a significant gap in clinical research evaluating how fluctuations in tryptophan-derived metabolites relate to acute postoperative pain. Although chronic pain associations have been explored, the relevance of tryptophan metabolites in acute surgical pain remains poorly defined. A recent review highlighted the critical role of tryptophan metabolism in modulating inflammation and neuronal activity—both central to pain processing—underscoring its potential clinical implications in acute pain contexts.^[12]^

Therefore, this observational study aimed to explore associations between circulating tryptophan metabolites and postoperative pain intensity, as measured by VAS, in patients undergoing surgery. By doing so, we sought to generate preliminary clinical evidence on the potential of these metabolites as biomarkers for pain perception in the perioperative setting.

## 2. Materials and methods

### 2.1. Patient selection and ethical approval

This observational study included 37 patients who underwent lumbar disc herniation surgery at the Dokuz Eylul University Faculty of Medicine, Department of Neurosurgery. The inclusion criteria were: patients aged 18 years or older, having provided informed consent, and undergoing a surgery expected to last at least 2 hours. Exclusion criteria included previous abdominal surgery, antibiotic use for intestinal conditions within the past 3 weeks, and known gastrointestinal disorders that may alter gut microbiota (e.g., Crohn disease, ulcerative colitis, and celiac disease).

The study was approved by the Dokuz Eylul University Hospital Non-Interventional Research Ethics Committee (Approval No: 7131-GO, dated August 31, 2022), and was registered at ClinicalTrials.gov (NCT06502223). All participants signed informed consent forms prior to enrollment.

### 2.2. Pain assessment (VAS)

Pain intensity was assessed using the VAS, a validated and widely used unidimensional tool for measuring subjective pain. The VAS consists of a horizontal 10 cm line, with endpoints labeled “no pain” (0) and “worst imaginable pain” (10). Patients marked a point on the line that best represented their current pain level, which was then measured in millimeters to produce a numerical value between 0 and 10.^[13]^

In this study, VAS scores were recorded at 3 time points: before surgery, and at 8 and 24 hours postoperatively. All assessments were conducted by trained healthcare professionals who were blinded to laboratory results to prevent bias. To avoid pharmacological interference, measurements were taken prior to the administration of rescue analgesics. This standardized method was applied to all participants to ensure consistency and reliability across evaluations.^[14]^

### 2.3. Blood sampling and processing

Venous blood samples (2 mL each) were collected from patients at 3 time points: preoperatively, 8 hours postoperatively, and 24 hours postoperatively. All blood draws were performed as part of routine perioperative care; no additional invasive procedures were required.

Samples were immediately centrifuged at 4000 rpm for 10 minutes, and the resulting serum and plasma fractions were transferred to labeled Eppendorf tubes and stored at −80°C until analysis. Each sample was paired with the corresponding VAS score and clinical parameters (pain descriptors and vital signs) for analysis.

### 2.4. LC–MS/MS metabolite analysis

Concentrations of tryptophan pathway metabolites (picolinic acid, 3-hydroxykynurenine, anthranilic acid, kynurenine, quinolinic acid, kynurenic acid, and xanthurenic acid) were measured using an Ultivo tandem mass spectrometer (Agilent 6465B, USA) coupled with an Agilent high-performance liquid chromatography (HPLC) system. A Conformité Européenne-In Vitro Diagnostic (CE-IVD) certified commercial kit (Jasem Kynurenine Pathway Metabolites LC–MS/MS, Sem Laboratory Devices, Istanbul, Turkey) was used.

- Calibrator preparation: 100 µL calibrator, 50 µL internal standard, and 250 µL Reagent-1 were mixed and transferred into an HPLC vial.
- Sample preparation: 100 µL of serum or plasma sample was mixed with 50 µL of internal standard and 250 µL of Reagent-1. The mixture was vortexed for 5 seconds and centrifuged at 4500 rpm for 5 minutes. The supernatant was transferred into HPLC vials for injection.

### 2.5. Statistical methods

The distribution of numerical variables was assessed using the Shapiro–Wilk test. Normally distributed variables were expressed as mean ± standard deviation, while non-normally distributed variables were reported as median (Q1–Q3). Categorical data were presented as counts and percentages.

Group comparisons were conducted using the independent samples *t* test or Mann–Whitney *U* test, as appropriate. For multiple group comparisons, one-way ANOVA or Kruskal–Wallis tests were employed. Repeated measures data were analyzed using the Friedman test; if significant, post hoc Wilcoxon tests with Bonferroni correction were applied. Receiver operating characteristic analysis was used to assess diagnostic performance, with the Youden index used to determine optimal cutoff points.

Spearman rank correlation was used to evaluate relationships between continuous variables. A heatmap was generated using Python’s Seaborn library to visualize correlation coefficients. Multivariate linear regression was performed to assess the effects of potential confounders such as body mass index (BMI), age, surgery duration, anesthesia time, and presence of chronic disease on pain outcomes.

Sample size estimation was conducted using G*Power 3.1 software, applying a point-biserial correlation model with an alpha of 0.05, power of 80%, and effect size of 0.50, yielding a minimum sample requirement of 26 patients.^[15]^ All analyses were performed using IBM SPSS Statistics version 29.0 and Python.

## 3. Results

### 3.1. Participant characteristics

A total of 37 patients were included in the study. The mean age was 50.2 ± 11.8 years, and the mean BMI was 27.6 ± 2.9 kg/m^2^. The sex distribution was 51.4% male and 48.6% female. Comorbidities included hypertension (24.3%), prior COVID-19 infection (18.9%), coronary artery disease (13.5%), diabetes (8.1%), and COPD (5.4%). All patients underwent lumbar disc herniation surgery under intravenous anesthesia induction followed by inhalational maintenance. The mean operation time was 144.3 ± 27.7 minutes and the mean anesthesia duration was 161.4 ± 31.5 minutes. Anesthetic agents administered during induction included propofol, midazolam, fentanyl, rocuronium, lidocaine, dexamethasone, ketamine, and paracetamol (Table 1). Non-steroidal anti-inflammatory drugs were included in postoperative analgesia in 62.2% of cases.

**Table 1:** Characteristics of patients and anesthesia induction parameters (n = 37).

Variables	Average ± SD
Age (yr)	50.2 ± 11.8
BMI (kg/m^2^)	27.6 ± 2.9
Propofol (mg)	195.4 ± 44.2
Fentanyl (mcg)	199.2 ± 56.7
Midozolam (mg)	1.5 ± 0.6
Rocuronium (mg)	44.6 ± 7.2
Lidocaine (2%) (mg)	70.5 ± 31.3
Dexamethasone (mg)	6.4 ± 3.2
Paracetamol (g)	0.9 ± 0.3
Ketamine (mg)	5.5 ± 12.8
Operation time (min)	144.3 ± 27.7
Anesthesia Time (min)	161.4 ± 31.5

This table presents the demographic data, anesthetic drug dosages, and durations related to the anesthesia and surgical procedures for 37 patients. The variables include the mean ± standard deviation (SD) for age, body mass index (BMI), the dosages of anesthetic agents used during induction, operation time, and total anesthesia duration. This comprehensive summary provides an overview of the patient characteristics and procedural details pertinent to the study cohort.

### 3.2. Pain outcomes

The preoperative median VAS score was 5.5 (2.75–8.25) for females and 6 (2–7) for males. At the 24th postoperative hour, VAS scores were significantly higher in males (*P* = .046). Pain types included stinging (32.4%), dull (21.6%), aching (13.5%), pressing (10.8%), and burning (10.8%).

Subgroup analysis revealed no significant differences in VAS score changes across sex, BMI categories, or presence of chronic disease (all *P* > .05). Multivariate analysis confirmed no significant effects of BMI (β = 0.091, *P* = .080), age (β = 0.015, *P* = .246), surgery duration (β = −0.035, *P* = .244), anesthesia duration (β = 0.018, *P* = .481), or chronic disease status (β = 0.023, *P* = .572) on pain outcomes.

### 3.3. Metabolite changes

Significant changes in tryptophan metabolite levels were observed for picolinic acid (*P* = .001), kynurenic acid (*P* = .006), and xanthurenic acid (*P* < .001) over the 3 time points (Table 2).

**Table 2:** Statistical analysis of tryptophan and its metabolites with effect sizes and confidence intervals.

Metabolite	Preoperative	Postoperative 8th hour	Postoperative 24th hour	*P*-value	Effect size (*d*)	95% Confidence interval
Tryptophan (x±s)	6810.6 ± 2212.1	6219.4 ± 1969.1	6805.2 ± 2403.1	.271	**0.29**	0.08–0.50
Picolinic acid*	1.45 (1.13–1.6)	1.09 (0.84–1.43)	1.00 (0.79–1.58)	.001	**N/A** (median)	N/A
3-OH kynurenine*	4.2 (2.65–6.49)	3.63 (2.56–5.5)	3.75 (2.56–6.94)	.649	**N/A** (median)	N/A
Anthranilic acid*	1.45 (1.01–2.09)	1.56 (1.17–2.07)	1.33 (1.02–2.09)	.110	**N/A** (median)	N/A
Kynurenine*	357.33 (268.25–482.92)	413.9 (301.81–520.15)	432.91 (316.7–528.84)	.245	**N/A** (median)	N/A
Quinolinic acid*	4.41 (1.95–7.03)	3.75 (1.85–5.82)	4.57 (2.88–12.18)	.158	**N/A** (median)	N/A
Kynurenic acid*	5.99 (4.25–8)	8.34 (5.14–11.36)	6.71 (5.12–9.51)	.006	**N/A** (median)	N/A
Xanthurienic acid*	1.8 (1.25–2.1)	1.2 (1–2.15)	1.00 (0.75–1.45)	<.001	**N/A** (median)	N/A

This table summarizes the statistical analysis of tryptophan and its metabolites measured at 3 time points: preoperative, postoperative 8th hour, and postoperative 24th hour. Tryptophan is presented as mean ± standard deviation (SD) and was analyzed using repeated measures ANOVA. Other metabolites are presented as medians (Q1–Q3) and were analyzed using the Friedman test. Effect sizes and 95% confidence intervals were calculated for the tryptophan values (*d* = 0.29; 95% confidence interval: 0.08–0.50) due to its normal distribution. For non-normally distributed metabolites, effect sizes were not calculated. Pairwise comparisons for significant metabolites were performed using the Wilcoxon test, with Bonferroni correction applied for multiple comparisons. Median values are given in bold, significance was examined using Friedman test. *Picolinic acid, 3‐OH kynurene, antralinic acid, kynurenine, quinolinic acid, kynurenic Acid, and xanthurenic acid preoperative, postoperative 8th‐hour and 24th‐hour values.

- Picolinic acid decreased from 1.45 (1.13–1.6) preoperatively to 1.09 (0.84–1.43) at 8 hours and 1.00 (0.79–1.58) at 24 hours.
- Kynurenic acid increased to 8.34 (5.14–11.36) at 8 hours, then decreased to 6.71 (5.12–9.51) at 24 hours, from a preoperative value of 5.99 (4.25–8.00).
- Xanthurenic acid decreased from 1.8 (1.25–2.1) to 1.2 (1–2.15) to 1.0 (0.75–1.45) over time.

Pairwise comparisons showed statistically significant differences between preoperative and 8-hour and 24-hour values for picolinic acid (*P* = .004 and *P* = .008, respectively). Xanthurenic acid showed significance between preoperative–24-hour (*P* = .001) and 8-hour to 24-hour (*P* = .023) (Table 3). Kynurenic acid showed a significant increase from preoperative to 8 hours (*P* = .001), but other comparisons were not significant.

**Table 3:** Pairwise comparisons with effect sizes and confidence intervals.

Metabolite	Δ1 (Preoperative to postoperative 8th hour)	Δ2 (Preoperative to postoperative 24th hour)	Δ3 (Postoperative 8th to postoperative 24th hour)	Effect size (*r*)	95% Confidence interval
Picolinic acid (*P*)	.004	.008	.360	.42	.18–.58
Kynurenic acid (*P*)	.001	.192	.126	.45	.21–.60
Xanthurienic acid (*P*)	.068	.001	.023	.39	.15–.55

This table displays the pairwise comparisons of picolinic acid, kynurenic acid, and xanthurenic acid levels across 3 time intervals: preoperative to postoperative 8th hour (Δ1), preoperative to postoperative 24th hour (Δ2), and postoperative 8th hour to postoperative 24th hour (Δ3). Pairwise comparisons were conducted using the Wilcoxon test. Effect sizes (*r*) and their 95% confidence intervals were calculated for the significant differences observed between the measurement points.

### 3.4. Correlation analyses

Correlation analysis revealed a significant association between 24-hour VAS score and quinolinic acid levels (*r* = 0.422, *P* = .009) (Table 4). No other tryptophan metabolites correlated with VAS at any time point.

**Table 4:** Correlation between tryptophan, its metabolites, and visual analog scale (VAS) pain scores at preoperative, postoperative 8th, and 24th hours.

Metabolit	Preoperative VAS (*r*)	Preoperative VAS (*P*)	Postoperative 8. Saat VAS (*r*)	Postoperative 8. Chaat VAS (*P*)	Postoperative 24. Saat VAS (*r*)	Postoperative 24. Chaat VAS (*P*)
Triptofan	0.189	.264	0.322	.052	0.015	.932
Picolinic acid	0.088	.604	‐0.168	.32	‐0.192	.255
3-OH kynurenine	‐0.156	.358	0.223	.185	0.118	.486
Anthralinic acid	0.059	.727	0.19	.261	0.280	.093
Kynurenine	‐0.151	.373	0.181	.285	0.023	.895
Quinolinic acid	‐0.138	.414	0.136	.423	0.422	.009
Kynurenic acid	0.037	.826	0.257	.124	0.101	.553
Xanthurienic acid	0.096	.571	0.184	.276	0.020	.907

This table presents the results of Spearman correlation analysis between tryptophan, its metabolites, and VAS pain scores measured preoperatively, at the postoperative 8th hour, and at the postoperative 24th hour. Correlation coefficients (*r*) and corresponding *P*-values (*P*) are shown for each metabolite across all time points. Statistically significant correlations (*P* < .05) are highlighted, indicating potential associations between specific metabolites and pain perception at various stages of the perioperative period.

**Figure 1.**
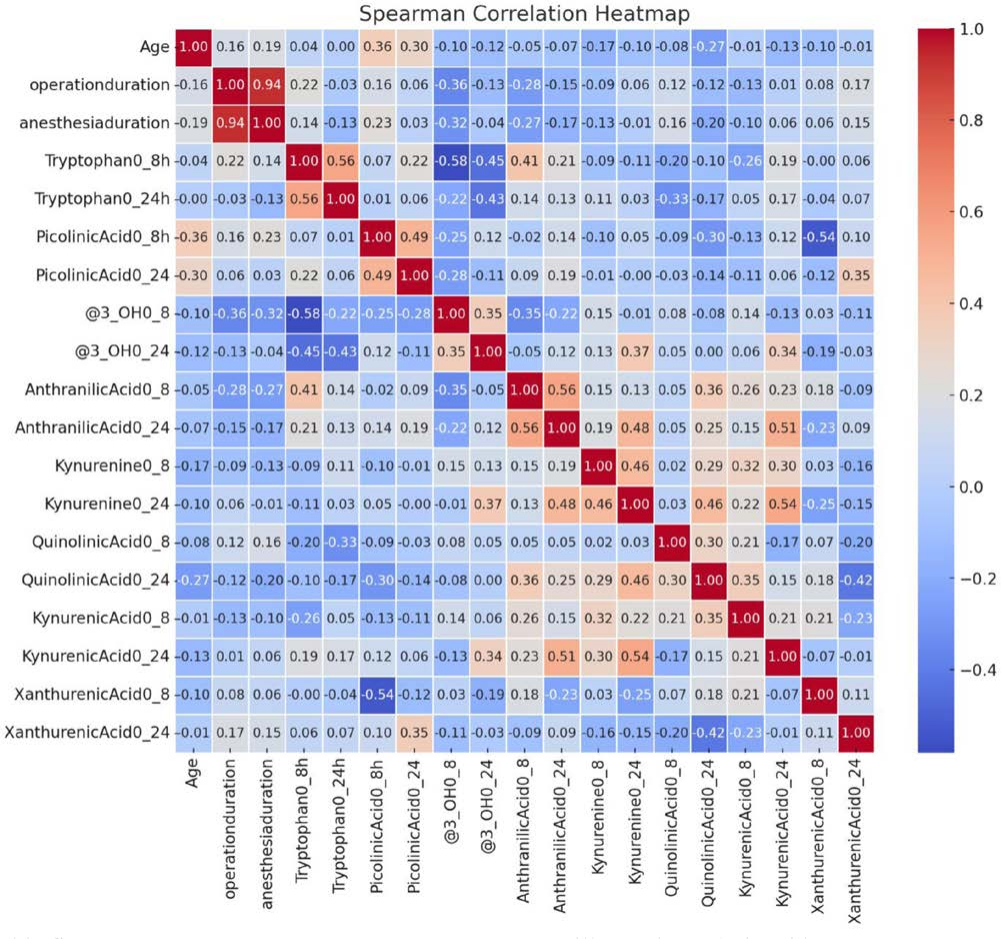
Correlation diagram between tryptophan, its metabolites, and clinical variables (age, operation time, and anesthesia time). This figure presents a Spearman correlation matrix illustrating relationships between tryptophan, its metabolites, age, operation time, and anesthesia duration. Statistically significant correlations (*P* < .05) are highlighted, with red indicating positive correlations and blue indicating negative correlations. Notably, age showed a weak positive correlation with Δpicolinic acid (*r* = 0.357; *P* = .030), while operation time had a weak negative correlation with Δ3-OH kynurenine (*r* = −0.359; *P* = .029). These findings suggest a potential link between tryptophan metabolism and perioperative biochemical changes.

In addition, operation time showed a weak negative correlation with the change in 3-OH kynurenine from preoperative to 8 hours (*r* = −0.359, *P* = .029), and age showed a weak positive correlation with the change in picolinic acid over the same time interval (*r* = 0.357, *P* = .030) (Fig. 1).

## 4. Discussion

The relationship between the microbiota and pain is a rapidly growing area of research. Despite the extensive literature on the microbiota and pain, studies specifically investigating the direct correlation between microbiota-derived metabolites in the bloodstream and acute pain remain limited. Thus, our study aimed to evaluate the potential relationship between acute pain and microbiota metabolite levels in blood.^[16,17]^

When examining tryptophan and its blood metabolites, significant changes were observed in the picolinic acid, kynurenic acid, and xanthurenic acid levels. Specifically, a marked decrease in picolinic acid level was detected during the postoperative period. However, this decrease did not appear to have a statistically significant association with pain perception.

In a study conducted in 2022, Shiro et al explored the relationship between sex, acute pain, and microbiota.^[18]^ Their research focused on microbiota phyla, specifically evaluating females, and identified Bacteroidetes, Firmicutes, Actinobacteria, and Proteobacteria as the main phyla. They found no significant changes in acute pain perception due to these phylum-level changes.

These findings suggest that exploring the gender-specific impact of microbiota on acute pain may require alternative approaches and larger-scale studies. Additionally, the use of blood metabolites instead of fecal samples allows for easier sampling, facilitating broader research. Our results suggest a possible association between tryptophan metabolism and postoperative pain perception. Specifically, the correlation observed between quinolinic acid levels and postoperative pain scores highlights a potential link that warrants further exploration in clinical research.

In contrast, our study investigated acute pain in relation to blood metabolite levels rather than stool-derived phylum changes. Furthermore, we included both male and female participants and found no gender differences in tryptophan metabolites.

Tryptophan metabolism is intrinsically linked to the kynurenine pathway, which regulates the balance between neurotoxic (quinolinic acid) and neuroprotective (kynurenic acid) metabolites. Quinolinic acid is a well-established N-methyl-d-aspartate receptor agonist, contributing to central sensitization and persistent pain states. Conversely, kynurenic acid acts as an N-methyl-d-aspartate receptor antagonist, potentially exerting neuroprotective and analgesic effects.^[19]^

These exploratory findings warrant further investigation into the mechanistic links between tryptophan metabolism and pain sensitivity. In particular, the potential role of indoleamine 2,3-dioxygenase modulation or microbiota-targeted therapies in influencing postoperative pain responses should be examined in longitudinal or interventional studies.^[20]^

Age-related changes in microbiota are well-established. Liufu et al demonstrated that anesthesia and surgery induce age-related alterations in the intestinal microbiome, particularly a decrease in the Lactobacillus phylum.^[21]^ Similarly, a meta-analysis by Badal et al revealed that the alpha diversity of microbial taxa, functional pathways, and metabolites was higher in older adults.^[22]^

In our study, the most notable age-related finding was a weak positive correlation between age and Δpicolinic acid levels (preoperative to postoperative 8-hour change; *r* = 0.357, *P* = .030). Instead of focusing on alpha and beta diversity, we examined postoperative metabolite differences. No significant correlations were observed between age and other tryptophan metabolites, including Δ3-OH kynurenine, Δanthranilic acid, Δkynurenine, Δquinolinic acid, Δkynurenic acid, or Δxanthurenic acid. These results underline the need for further exploration of age-related changes in the microbiota and metabolites.

Guo et al evaluated the effects of propofol on the intestinal microbiota and found that continuous intravenous propofol infusion minimally impacted the gut flora in rats.^[23]^ However, their study was limited to infusion-based administration. In our study, propofol was used during induction at a standard dosage (195.4 ± 44.2 mg), but not as a continuous infusion, restricting our ability to assess its impact on the microbiota.

In our study, maintenance anesthesia was induced using sevoflurane. Previous research, including that by Han et al,^[24]^ has demonstrated that sevoflurane inhalation anesthesia can cause alterations in the intestinal microbiome. However, in our study, no significant correlations were found between tryptophan metabolite levels and the operation duration. This might be due to the use of inhalation anesthesia, which could influence microbiota evaluation. Future studies incorporating direct gut microbiota analysis are needed to validate these findings.

In contrast to our study of acute pain, Gunn et al investigated chronic pain using a biomarker test panel. This panel evaluates systemic inflammation, oxidative stress, neurotransmitter cycling, and microbiota status. Studies on chronic pain aim to identify precise biomarkers and develop targeted treatment strategies.^[25]^ However, more recent work on blood-based metabolomics in acute settings aligns with our study’s scope.^[26]^

In our study, significant changes were observed in the quinolinic acid levels, which decreased postoperatively at 8 hours but increased at 24 hours. Unlike chronic pain biomarkers, such as methylmalonic acid or homocysteine, our findings focused on acute pain and microbiota-related metabolites.

## 5. Strengths and limitations

This study is among the first to correlate dynamic changes in blood tryptophan metabolites with acute postoperative pain in a lumbar surgery cohort. Strengths include its focus on a well-defined surgical population, structured timing of sample collection, and integration of biomarker analysis with clinical pain assessments. Moreover, blood-based analysis allowed for repeated and standardized sampling across all time points.

Limitations include the relatively small sample size and the absence of stool-based microbial profiling, which would have enabled taxonomic or functional correlation. Future studies should integrate metabolomic and microbiota data using advanced omics tools such as 16S rRNA and metagenomics. Moreover, larger, multicenter trials could improve the generalizability and statistical power of the results.

## 6. Conclusion

Our study contributes novel insights by focusing on blood-derived microbiota metabolites in the context of acute pain. The use of serum samples rather than stool provides a practical, less invasive improves the generalizability of our results.

However, this study is limited by its modest sample size and lack of direct microbiota composition analysis. Future research should incorporate fecal 16S rRNA or metagenomic sequencing to directly associate microbial taxa with metabolite shifts and pain responses. Broader biomarker panels, beyond tryptophan metabolites, may also help clarify the complex mechanisms underlying postoperative pain.

### Acknowledgments

The authors express their sincere gratitude to the late Ahmet Çelik, father and first teacher of the corresponding author, whose lifelong dedication and values continue to inspire this work. This study is respectfully dedicated to his guiding principle: *“The most beneficial person to humanity is the one who performs their work best.”* The authors also acknowledge the valuable academic mentorship and scientific support provided by the Department of Anesthesiology and Reanimation at Dokuz Eylul University.

## Author contributions

**Conceptualization:** Yunus Celik, Hale Aksu, Ferim Sakize Gunenc, Orhan Kalemci.

**Data curation:** Hale Aksu, Orhan Kalemci, Ceren Kizmazoglu, Pembe Keskinoglu.

**Formal analysis:** Ceren Kizmazoglu, Pembe Keskinoglu.

**Investigation:** Hale Aksu, Ferim Sakize Gunenc.

**Methodology:** Yunus Celik, Hale Aksu, Pembe Keskinoglu.

**Project administration:** Yunus Celik.

**Supervision:** Hale Aksu.

**Visualization:** Hale Aksu, Orhan Kalemci.

**Writing – original draft:** Yunus Celik, Hale Aksu.

**Writing – review & editing:** Yunus Celik, Hale Aksu.
